# Ultrasound guided high intensity focused ultrasound (USgHIFU) for malignant tumors: survival advantage in stage III and IV pancreatic cancer.

**DOI:** 10.1186/2050-5736-3-S1-O79

**Published:** 2015-06-30

**Authors:** Joan Vidal-Jove, Eloi Perich, Angels Jaen, Alvarez del Castillo Manuel

**Affiliations:** 1 Hospital University Mutua Terrassa, Terrassa, Spain

## Background/introduction

We describe the experience of the HIFU Surgical Oncology Unit of Hospital University Mutua Terrassa (Barcelona, Spain) treating malignant tumors. We analyze results in unresectable pancreatic tumors treated with USgHIFU hyperthermic ablation plus adjuvant chemotherapy.

## Methods

From February 2008 to April 2014 we have treated 148 malignant cases. Of those, 48 cases of non resectable pancreatic tumors were treated from March 2010 to April 2014, and we include the first 43 patients to the analysis (until December 2013). All of them underwent systemic chemotherapy with a standard combination. Clinical responses (thermical ablation achieved) were measured by image techniques. They were 29 Stage III cases and 14 Stage IV cases. Complications were also analyzed.

## Results and conclusions

The distribution of the 148 cases treated reflects a majority of pancreatic and liver tumors. We analyze the 43 pancreatic tumors. Clinical responses (ablation obtained) were 82% in all cases, sustained at 8 weeks of the procedure. We obtained 11 complete responses (25%) at the end of the combined treatment, 9 from stage III patients and 2 from stage IV. Major complications included severe pancreatitis with GI bleeding (1), skin burning grade III that required plastic surgery (2). No deaths due to the procedure were registered. Overall Median Survival is 16 month (6 mo – 3.4 year) Conclusions: HIFU is a potentially effective and safe modality for the treatment of malignant tumors. Compared with published data, HIFU proves survival advantage in non resectable stage III and IV pancreatic cancer.

